# Tale of cAMP as a second messenger in auxin signaling and beyond

**DOI:** 10.1111/nph.19123

**Published:** 2023-07-11

**Authors:** Linlin Qi, Jiří Friml

**Affiliations:** ^1^ Institute of Science and Technology Austria (ISTA) Klosterneuburg 3400 Austria

**Keywords:** adenylate cyclase, auxin signaling, cyclic adenosine monophosphate (cAMP), second messenger, transport inhibitor response 1/auxin‐signaling F‐box proteins (TIR1/AFBs)

## Abstract

The 3′,5′‐cyclic adenosine monophosphate (cAMP) is a versatile second messenger in many mammalian signaling pathways. However, its role in plants remains not well‐recognized. Recent discovery of adenylate cyclase (AC) activity for transport inhibitor response 1/auxin‐signaling F‐box proteins (TIR1/AFB) auxin receptors and the demonstration of its importance for canonical auxin signaling put plant cAMP research back into spotlight. This insight briefly summarizes the well‐established cAMP signaling pathways in mammalian cells and describes the turbulent and controversial history of plant cAMP research highlighting the major progress and the unresolved points. We also briefly review the current paradigm of auxin signaling to provide a background for the discussion on the AC activity of TIR1/AFB auxin receptors and its potential role in transcriptional auxin signaling as well as impact of these discoveries on plant cAMP research in general.

**Table:** 

	Contents	
	Summary	489
I.	Introduction	489
II.	cAMP in mammalian cells: well‐known second messenger in signal transduction	490
III.	Brief and controversial history of plant cAMP research	490
IV.	TIR1/AFBs‐mediated auxin signaling: canonical transcriptional reprogramming and enigmatic rapid nontranscriptional responses	492
V.	Discovery of the AC activity for TIR1/AFB auxin receptors supports cAMP as the second messenger in canonical auxin signaling	492
VI.	Conclusions	493
	Acknowledgements	494
	References	494

## I. Introduction

**Fig. 1:**
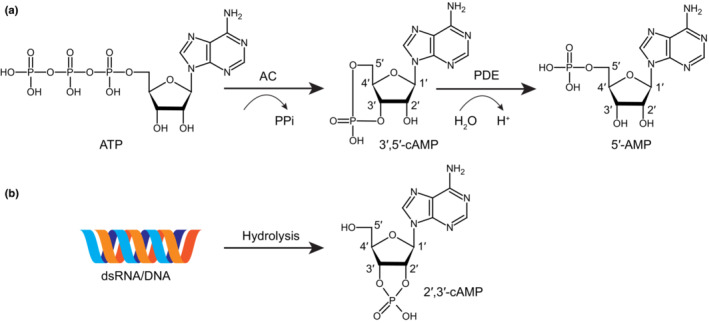
Chemical structure and homeostasis of cyclic nucleotide monophosphates (cNMPs). (a) Structure and homeostasis of 3′,5′‐cyclic adenosine monophosphate (cAMP). 3′,5′‐cAMP is produced from adenosine triphosphate (ATP) by adenylate cyclase (AC) and can be further hydrolyzed to adenosine 5'‐monophosphate (5′‐AMP) by cNMP phosphodiesterases (PDE). 3′,5′‐cGMP is produced from guanosine triphosphate (GTP) by guanylate cyclase (GC) and is hydrolyzed by PDE in a similar way. (b) Biogenesis of 2′,3′‐cAMP. 2′,3′‐cAMP and 2′,3′‐cGMP are produced during the hydrolysis of dsRNA/DNA.

The cyclic nucleotide monophosphates (cNMPs) are single‐phosphate nucleotides with a cyclic bond between the sugar and phosphate groups. According to the different linkage positions of the cyclic bond, there are two naturally occurring isoforms: 3′,5′‐cNMP and 2′,3′‐cNMP (Fig. 1). The well‐studied and commonly referred one is 3′,5′‐cNMP. Especially, 3′,5′‐cyclic adenosine monophosphate (cAMP) and 3′,5′‐cyclic guanosine monophosphate (cGMP) are well‐known second messengers produced from adenosine triphosphate (ATP) by adenylate cyclase (AC) or from guanosine triphosphate (GTP) by guanylate cyclase (GC), respectively. The cellular cAMP/cGMP can also be hydrolyzed by cNMP phosphodiesterases (PDE) controlling their homeostasis (Fig. 1; Beavo & Brunton, 2002). The atypical 2′,3′‐cNMPs detected in different organisms have been shown to be derived from DNA/RNA degradation. They have less‐documented roles in signaling as compared to the typical 3′,5′‐cNMP, but has proved to be generated during and involved in organ injury and immune responses (Jackson, 2011; Yu *et al*., 2022).

## II. cAMP in mammalian cells: well‐known second messenger in signal transduction

**Fig. 2:**
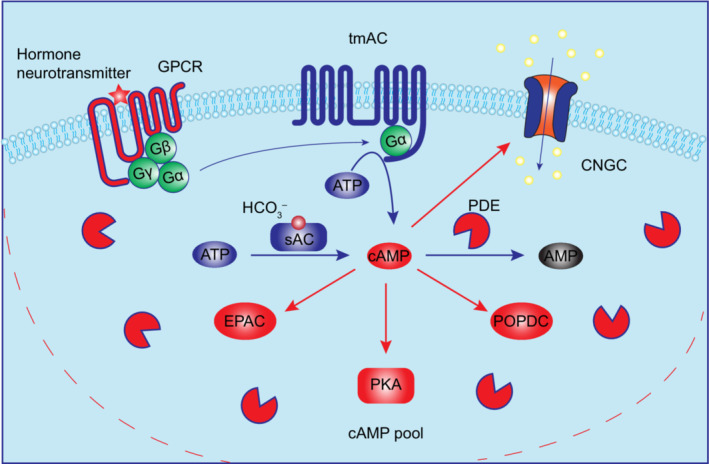
Cyclic adenosine monophosphate (cAMP) signaling in mammalian cells. Signal molecule like hormone or neurotransmitter binds to G protein‐coupled receptor (GPCR), and the associated heterotrimeric G protein is activated. The released Gα subunit binds and activates transmembrane adenylate cyclase (tmACs) to produce cAMP. Soluble AC (sAC) in cytoplasm can be activated by bicarbonate anions (HCO_3_^−^) to produce cAMP. cAMP mediates various downstream responses through directly binding its downstream effectors and regulating their function. The well‐characterized cAMP downstream effectors include protein kinase A (PKA), cyclic nucleotide‐gated channels (CNGC), exchange protein activated by cAMP (EPAC) and Popeye domain containing proteins (POPDC). cAMP can be further hydrolyzed by PDE. Efficient cAMP hydrolysis by phosphodiesterases (PDEs) in vicinity restricts the cAMP diffusion within cells leading to formation of spatially restricted subcellular local cAMP pool. This is possibly the key ingredient assuring specificity of signal transduction. ATP, adenosine triphosphate.

Since its original discovery in 1958 by Earl W. Sutherland that cAMP plays an intermediary role in glycogen breakdown triggered by the mammalian hormone epinephrine (Nicole *et al*., 2005), eventually this single molecule has been shown to be crucial and versatile second messenger involved in many different signaling pathways (Beavo & Brunton, 2002). Generally, extracellular ligands like hormone or neurotransmitter binds and activates heterotrimeric G protein (Gα, Gβ and Gγ subunits)‐coupled receptor (GPCR). This leads to release of the Gα subunit, which can further bind and activate transmembrane AC (tmAC) to produce cAMP. cAMP directly binds its downstream effectors and regulates their functions to trigger different cellular responses in different physiological context. The well‐established cAMP effectors in mammalian cells include protein kinase A (PKA), exchange protein activated by cAMP (EPAC), cyclic nucleotide‐gated channels (CNGC) and Popeye domain‐containing proteins (POPDC) (Fig. 2; Ostrom *et al*., 2022). An exception is the soluble AC (sAC) involved in male fertility, which is localized in cytoplasm and cellular organelles and is uniquely regulated by bicarbonate anions (Tresguerres *et al*., 2011).

In human genome, there are *c*. 335 seven transmembrane (7TM) receptors in the GPCR superfamily, 20 Gα subunits and 10 ACs (9 tmACs and 1sAC; Beavo & Brunton, 2002). They have different expression pattern and different combinations in mediating signal transduction (Ostrom *et al*., 2022). Nonetheless, even in a specific cell type, dozens of signaling pathways use the same second messenger cAMP to trigger diverse cellular responses, which was first, when Sutherland originally proposed the hypothesis, met with a strong criticism as it was difficult to imagine how the signal > response specificity could be assured while using the same intermediate messenger. Now, the concept of ‘cAMP signaling compartmentation’ was proposed to address this issue. Simply, within the cell, the specific AC at the center, receptor and its downstream effectors are spatially close to each other forming subcellular signaling compartment. Certain PDE isoforms in the same compartment will hydrolyze cAMP to restrain its diffusion, forming subcellular cAMP pool, so that cAMP generated can only specifically activate the effectors in the close vicinity, that is, in the same compartment (Fig. 2). In this way, a common second messenger cAMP can specifically mediate different cellular responses after perception of different signals (Stangherlin & Zaccolo, 2012; Johnstone *et al*., 2018).

## III. Brief and controversial history of plant cAMP research

After the 1971 Nobel Prize in medicine or physiology was awarded to Sutherland for his discovery of cAMP as the second messenger in mammals, it attracted the attention and interest of plant scientists, and in the 1970s and 1980s, plant cAMP research seemed to boom. The results were, however, quite controversial or even contradictory. cAMP was reported to be detected in various organs, tissues of plant species (Amrhein & Filner, 1973; Ashton & Polya, 1978; Johnson *et al*., 1981), but other reports were skeptical about the vanishingly small quantities, interfering substances in plant tissues, potential contaminants from microbes during sample preparation and about unspecific or less reliable detection methods (Spiteri *et al*., 1989). Meanwhile, by treating plants with those well‐established tools from mammalian research, like cell‐permeable cAMP, AC activators, or PDE inhibitors, various physiological responses were reported (Levi *et al*., 1981; Chopra & Sharma, 1985), but other accounts reported no effects (Edgerton *et al*., 1975). Moreover, AC and PDE activities were also detected in plant extracts (Aline *et al*., 1984; Martelli *et al*., 1987), but when after > 20 yr of plant cAMP research since the first report in 1973, no AC had been successfully identified, a dogma started to prevail that higher plants do not possess ACs and do not use cAMP for signaling and are thus unique among kingdoms of living organisms (Trewavas, 1997). Consequently, plant cAMP research declined in the 1990s, not least because the booming forward genetic approaches of that era failed to identify any obvious candidates for cAMP‐related components.

The doubts about the presence of cAMP in plants were finally resolved by its positive identification thanks to the improvements of chromatography and the advent of mass spectrometry (Newton, 1996), though skeptics are still arguing with its low levels in plant tissues. The first plant AC candidate (*axi141*) was identified by screening of the activation T‐DNA tagged tobacco cell lines that could proliferate in the absence of auxin, supporting an important role of cAMP in auxin signaling (Ichikawa *et al*., 1997). This seemed to be a breakthrough in the field as also put by the Nature News and Views ‘Plant cyclic AMP comes in from the cold’ (Trewavas, 1997), which accompanied the original research article. Unfortunately, the paper was retracted the following year due to the fact that the key result related to cAMP‐dependent protoplast division in the absence of auxin was not correct (Ichikawa *et al*., 1998), which sent the field again into the ice age.

After this rather calamitous event, only few scientists continued working on plant cAMP. Moreover, when the *Arabidopsis* genome was released in 2000 (Arabidopsis Genome Initiative, 2000), it appeared that *Arabidopsis* proteome lacks orthologous to the mammalian AC families. However, in 2001, pollen‐signaling protein (PSiP) from *Zea mays* was isolated from a maize cDNA library, which showed homology to AC from fungi and was demonstrated to produce cAMP *in vitro* (Moutinho *et al*., 2001). Its putative homologs in *Arabidopsis* are nucleotide‐binding site‐leucine‐rich repeat (NBS‐LRR) disease‐resistance proteins, suggesting that plant ACs may only have short conserved core motif, which is combined with other specialized domains. To search for those disguised AC candidates, a conserved core AC motif ([RKS]X[DE]X{9,11}[KR]X{1,3}[DE]) was successfully extracted through aligning the sequences of catalytic centers of the well‐characterized ACs from mammals, fungi and prokaryotes, which is essential for catalysis and where [RKS] in position 1 allows hydrogen binding with adenine, [DE] in position 3 confers substrate specificity for ATP, [KR] stabilizes the transition state from ATP to cAMP, and the final [DE] residue is the cofactor Mg^2+^/Mn^2+^ binding site (Liu *et al*., 1997; Gehring, 2010). Searching *Arabidopsis* proteome with this motif or modified versions obtained the potential list of AC candidates. Biochemical characterization indeed confirmed that some of them showed AC activity, such as K^+^‐uptake permease 7 (AtKUP7; Al‐Younis *et al*., 2015) and (AtKUP5; Al‐Younis *et al*., 2018), clathrin assembly protein (AtClAP; Chatukuta *et al*., 2018), a leucine‐rich repeat protein (AtLRRAC1; Bianchet *et al*., 2019), the 9‐*cis*‐epoxycarotenoid dioxygenase (NCED3; Al‐Younis *et al*., 2021), a protein with unknown function AtAC (Sehlabane *et al*., 2022), and the list continues growing in recent years. However, most of those reports focused on the biochemical characterization of their *in vitro* AC activity, and their importance for signaling and physiology *in planta* was not fully addressed or well‐recognized. The unexpected discovery of the AC activity for the TIR1/AFBs auxin receptors along with its crucial importance for the canonical auxin signaling (Qi *et al*., 2022) provided so far the most complete demonstration of the AC role in plant signaling, potentially bringing plant cAMP research back into spotlight.

In short, when we trace back the 50 yr dramatic history of plant cAMP research, major progress has been achieved: (1) Presence of cAMP in plants had been fully confirmed; (2) Quite some plant ACs were identified. Unlike mammalian AC families, plant ACs are mostly moonlighting proteins, with core AC motif combined with other divergent functional domains (Gehring & Turek, 2017); (3) Increasing evidence indicates that cAMP is involved in many physiological processes in plants, such as ion homeostasis, stomata opening, pollen tube growth, seed germination, cell cycle progression, abiotic stress responses and plant innate immunity (Blanco *et al*., 2020).

Nonetheless, there are still important issues preventing the full acceptance of cAMP as a second messenger by the plant community: (1) cAMP levels in plants are lower than in animals despite direct comparisons are problematic. This may be explained by the hypothesis that cAMP in plant cell is more strongly concentrated locally due to a higher level of compartmentation, supported by the presence of dual‐domain AC/PDE recently reported in plants (Kwiatkowski *et al*., 2021; Hayashida *et al*., 2022), but so far, there is no direct evidence to support this; (2) Convincing genetic evidence for the importance of plant ACs for cell signaling is generally lacking. This may be also due to multifunctionality of most plant ACs. Thus, mutations in AC center often impairs also the function of the neighboring domain (Al‐Younis *et al*., 2018), making their decoupling very difficult; (3) Downstream effectors are elusive. Though the CNGC proteins are the most promising candidates in many cases (Jarratt‐Barnham *et al*., 2021) and a list of cAMP‐binding proteins was reported (Donaldson *et al*., 2016), none of them has been validated; (4) Technical complexities. Possibly due to a strong cAMP signaling compartmentation, cAMP mobility within cells may be very limited, which makes exogenous treatment with cell‐permeable cAMP in plants inefficient and typically inconclusive. Moreover, a usage of the AC activators or inhibitors characterized in mammalian research without knowing their effectiveness and specificity for the plant ACs made data interpretation complicated, especially when we now know plant ACs are much more divergent from mammalian ACs and have different activation mechanisms.

All those are serious arguments but with our continued efforts to uncover a fact or its best approximation, some of them may get clarified and others outweighed by new findings, finally reconciling them with a more positive view on the role of cAMP in plant signaling.

## IV. TIR1/AFBs‐mediated auxin signaling: canonical transcriptional reprogramming and enigmatic rapid nontranscriptional responses

Auxin is the pivotal hormone for plant growth and development (Friml, 2021), and thus, its signal transduction mechanism has always been of a great interest. Biochemical and molecular genetic approaches in *Arabidopsis* culminated in 2005 with realization that TIR1/AFBs are the nuclear auxin receptors mediating transcriptional reprogramming (Dharmasiri *et al*., 2005; Kepinski & Leyser, 2005). After the elucidation of the receptor complex structure (Tan *et al*., 2007), the so‐called canonical auxin signaling pathway seemed to be fully established and sufficient to explain the auxin‐induced transcriptional regulations. Briefly, TIR1/AFBs are F‐box proteins, the subunits determining the substrate specificity of the Skp1‐cullin 1‐F‐box (SCF) type E3 ubiquitin ligase complex. Auxin binding to TIR1/AFBs promotes its interaction with the auxin/indole‐3‐acetic acid (Aux/IAA) co‐receptors leading to their ubiquitination and degradation. Aux/IAAs also act as transcriptional repressors, which directly interact with the auxin response factor (ARF) transcription factors and repress their activity. After degradation of Aux/IAAs following auxin perception, ARF‐mediated transcriptional reprogramming is initiated. For more details, please see more comprehensive reviews on auxin signaling (Parry & Estelle, 2006; Quint & Gray, 2006; Gallei *et al*., 2020).

A great success of this TIR1/AFBs‐Aux/IAAs‐ARFs textbook model for explaining most of the auxin‐regulated developmental processes led to a decreased interest in rapid, nontranscriptional auxin responses, although they had been studied for decades well before the reign of genetics and molecular biology. The rapid auxin cellular responses include plasma membrane depolarization, apoplast alkalinization and cytosolic Ca^2+^ increase (Gallei *et al*., 2020; Friml, 2021). As TIR1/AFBs were linked exclusively with regulation of transcription, to explain these rapid auxin effects, existence of additional auxin receptors was invoked (Shih *et al*., 2015). However, recent evidence surprisingly revealed that many of these rapid effects actually also depend on the TIR1/AFB receptors (Dindas *et al*., 2018; Li *et al*., 2021), mainly on the cytosolic AFB1 (Serre *et al*., 2021). Additionally, the classical inhibitory auxin effect on root growth underlying gravitropic response, which was known to depend on TIR1/AFB receptors and Aux/IAA co‐receptors, was ultimately shown to be too fast to involve transcriptional regulation (Fendrych *et al*., 2018). These unexpected contradictions all implied an existence of a nontranscriptional branch downstream of TIR1/AFBs and prepared a stage for further unexpected discoveries in the canonical, TIR1/AFB‐mediated auxin signaling.

## V. Discovery of the AC activity for TIR1/AFB auxin receptors supports cAMP as the second messenger in canonical auxin signaling

While searching for the potential mechanism underlying the TIR1/AFB‐mediated rapid nontranscriptional responses, by a combination of educated reasoning and motif search, we discovered that TIR1/AFBs have AC activity (Qi *et al*., 2022). TIR1/AFBs have conserved domain structure. F‐box motif is in the N‐terminal, the middle major part is the leucine‐rich‐repeats (LRR), and in the short unannotated C‐terminal region, a conserved AC core motif was found. Complementation of an AC‐deficient *Escherichia coli* mutant strain and *in vitro* AC activity assays with purified proteins clearly showed that all the *Arabidopsis* TIR1/AFB proteins, which can be expressed and purified, have AC activity. The AC motif is highly conserved even in the basal land plant *Physcomitrium patens*, and all the 4 PpAFBs purified had a clear AC activity, indicating that it is conserved in all land plants. In the presence of the co‐receptors Aux/IAAs, auxin stimulated the AC activity of TIR1/AFBs *in vitro*, and auxin treatment also steadily increased cAMP levels in roots. Mutations in TIR1 AC domain specifically abolished the AC activity without affecting the auxin perception function of TIR1, that is, the auxin‐induced interaction with Aux/IAAs. Nonetheless, they severely compromised TIR1 function in mediating sustained root growth inhibition and auxin‐induced transcription strongly suggesting that TIR1 AC activity is crucial for the canonical auxin signaling (Qi *et al*., 2022). By contrast, loss of AC activity in TIR1 does not affect rapid auxin responses, including Ca^2+^ transients and apoplastic alkalinization, implying that TIR1 AC activity is not required for these rapid responses (Qi *et al*., 2022). Recently, it has been demonstrated that the predominantly cytoplasmic localized AFB1 plays major roles in mediating rapid auxin responses (Serre *et al*., 2021). Hence, it remains open whether AFB1 AC activity is involved in rapid auxin responses, even though it seems unlikely given that auxin treatment increases cAMP levels in roots only with a slow dynamics.

**Fig. 3:**
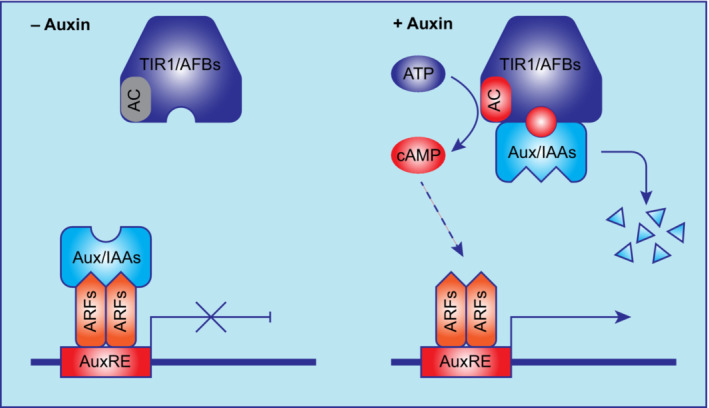
Cyclic adenosine monophosphate (cAMP) as the second messenger in auxin signaling. Without auxin, the adenylate cyclase (AC) activity of transport inhibitor response 1/auxin‐signaling F‐box proteins (TIR1/AFB) auxin receptors is low. The transcriptional repressors auxin/indole‐3‐acetic acid (Aux/IAAs) are stable, interact with and repress auxin response factor (ARF) transcriptional activators. Thus, auxin‐induced transcription is turned off. Auxin binding to TIR1/AFBs promotes their interaction with Aux/IAAs, leading to Aux/IAA's ubiquitination and degradation, thus releasing ARFs from their repression. Simultaneously, AC activity of TIR1/AFBs is enhanced to produce more cAMP after auxin perception, which is also crucial for the final transcriptional response. Hence, Aux/IAAs' degradation and cAMP production are two signaling outputs of the auxin perception, both required for the auxin‐induced transcription. ATP, adenosine triphosphate.

Originally, Aux/IAAs degradation was thought to be the only signaling output of the auxin receptors. These studies identified their other output – the cAMP production – an activity, which is unexpectedly indispensable for the transcriptional responses (Fig. 3). These observations advocate cAMP as the second messenger in canonical auxin signaling. However, it cannot be excluded that the used AC mutations affect also other properties or unknown functions of TIR1 and/or that cAMP does not act as a true second messenger but the AC activity and generated cAMP only influence locally TIR1 functions without targeting other proteins. Hence, the cAMP second messenger theory still awaits rigorous confirmation.

The discovery of AC activity for TIR1/AFB auxin receptors and its importance in canonical auxin signaling raises a few obvious questions: (1) Is the TIR1/AFBs' AC activity linked to the Aux/IAAs degradation? (2) Is the TIR1/AFB‐produced cAMP sufficient to induce downstream transcriptional responses? (3) What are the cAMP effectors downstream of auxin receptors, if any? ARFs, Aux/IAAs, or other, yet unknown components? Considering that the well‐known cAMP effectors in mammals are ion channels and kinases, it is possible that the Aux/IAAs‐ARF transcription module is regulated indirectly. (4) Do TIR1/AFB receptors have additional functionalities responsible for the rapid nontranscriptional auxin responses?

Clarification of those questions has potential to redefine or dramatically expand our current paradigm of auxin signaling.

## VI. Conclusions

Historically, plant cAMP research has been controversial for more than half a century. The importance of AC activity by TIR1/AFBs auxin receptors provides a support for the importance of cAMP in cell signaling in plants, which may become an impulse to rejuvenate the plant cAMP research. Previous physiological studies relying mainly on pharmacological manipulations suggested that cAMP is involved in various physiological responses (Gehring, 2010; Blanco *et al*., 2020), which will hopefully get further elucidated by complementary genetic studies and eventually help to establish cAMP as a versatile second messenger in various plant signaling pathways. Searching the plant genomes, the most components of the much‐studied cAMP circuit from mammals are not found: no GPCRs, no typical ACs and PKAs. Moreover, homology of plant ACs to mammalian AC isoforms is limited to only a short core catalytic center conserved, which also hampered its original identification. Thus, it seems that plant ACs and their product, cAMP, act differently in plants and animals. For example, plant ACs seem to be mostly moonlighting proteins, with the core AC motif combined with other divergent functional domains (Gehring & Turek, 2017). An interesting question is whether plant and mammalian ACs have a common origin and diverged dramatically afterward, or they arose independently during evolution.

After a slow start and serious set‐backs in the past, we are still writing only the first lines of the plant cAMP tale: (1) We still do not know how many ACs are hidden in plant proteomes, for example, whether other F‐box proteins also have similar AC activity, especially the closest homolog of TIR1/AFBs – the jasmonate receptor COI1; (2) thus, it remains unclear, in which other signaling pathways cAMP may be involved; (3) we do not know how they are inactivated, even though there were promising plant PDE candidates identified based on sequence of canonical PDE catalytic centers (Kwiatkowski *et al*., 2021; Hayashida *et al*., 2022); and (4) we do not know verified identities of cAMP effector proteins. But we can be sure that the cAMP tale will continue being dramatic, exciting, full of unexpected twists and with conclusions to remember.

## Competing interests

None declared.
